# Natural extract–derived compounds for the treatment of hematological diseases through modulation of the bone marrow microenvironment

**DOI:** 10.3389/fchem.2026.1790399

**Published:** 2026-03-09

**Authors:** Ningrui Wang, Ningning Zhu, Nana Zhou, Nanxi Dong, Jingjing Xiang, Baodong Ye, Jingjing Liu

**Affiliations:** 1 Department of Hematology, The First Affiliated Hospital of Zhejiang Chinese Medical University (Zhejiang Provincial Hospital of Chinese Medicine), Hangzhou, Zhejiang, China; 2 The First School of Clinical Medicine, Zhejiang Chinese Medical University, Hangzhou, Zhejiang, China; 3 Laboratory of Integrated Traditional Chinese and Western Medicine for Hematology, The First Affiliated Hospital of Zhejiang Chinese Medical University (Zhejiang Provincial Hospital of Chinese Medicine), Hangzhou, Zhejiang, China

**Keywords:** bone marrow microenvironment, drug design, hematological diseases, medicinal chemistry, natural extracts

## Abstract

The bone marrow microenvironment comprises a complex network of hematopoietic stem cells, immune cells, stromal cells, and non-cellular components such as the extracellular matrix and soluble factors, collectively maintaining the homeostasis of hematopoietic stem and progenitor cells. It is highly vulnerable to pathological perturbations induced by hematologic malignancies, solid tumors, inflammatory stress, and therapeutic exposure, which collectively destabilize microenvironmental homeostasis and promote hematopoietic failure, malignant progression, and immune dysregulation. Natural products exhibit unique advantages in modulating the blood microenvironment due to their structural diversity, multitarget effects, and low toxicity. Their biological activities span multiple mechanistic dimensions, including redox regulation, metal ion homeostasis, signaling inhibition, and microenvironment remodeling. However, the intrinsic relationships between their chemical structures and biological functions have not yet been systematically elucidated. Therefore, from a translational medicine perspective, this review focuses on elucidating the pharmacological mechanisms by which natural products regulate the hematopoietic microenvironment. We systematically summarize their chemical basis and structure–activity relationships, together with recent advances in extraction techniques, chemical modification, and targeted delivery strategies. The aim is to bridge the gap between chemical research on natural products and their clinical therapeutic applications, providing a framework and innovative directions for drug development targeting hematological diseases.

## 1 Introduction

The hematopoietic system is essential for maintaining physiological homeostasis and immune function. Its core regulatory mechanism lies in the precise control of the bone marrow hematopoietic microenvironment (Cossío et al., 2019). The bone marrow microenvironment (BME) comprises hematopoietic stem cells (HSCs), immune cells, stromal cells, growth factors, cytokines, and extracellular matrix components (Gong and Cheng, 2015). Dysregulation of the BME can lead to a series of bone marrow-related diseases, including leukemia, aplastic anemia, lymphoma, and myelodysplastic syndromes (Sheng et al., 2025). Additionally, malignant or dysfunctional cells actively secrete soluble factors such as CXCL12, IL-6, and IL-1β. These factors have been demonstrated to remodel the BME (Hou et al., 2025; Kojima et al., 2011), promoting the proliferation, migration, and adhesion of malignant cells. In parallel, it activates pathways associated with drug resistance (Xiong et al., 2025). Immunosuppression-driven tumor progression further alters the extracellular matrix. This process facilitates immune evasion and reinforces resistance barriers. As a result, disruption of the BME becomes more severe (Cui et al., 2024). Consequently, therapeutic regulation of the BME has emerged as a fundamental strategy for the treatment of hematological disorders.

Current treatments for hematologic diseases include hematopoietic stem cell transplantation, immunotherapy, chemotherapy, and supportive care. These approaches often cause systemic side effects. They also increase the risk of infection and drug resistance (Pham et al., 2025; Wang W. et al., 2025). Natural products offer unique advantages in the treatment of hematologic diseases and in the modulation of the BME. These advantages arise from their structural diversity and multitarget pharmacological actions (Iweala et al., 2023; Lin et al., 2020). Natural products include plant- and mineral-derived extracts from traditional medicines. Researchers widely recognize these compounds as adjuvants in chemotherapeutic treatments, which can improve therapeutic efficacy and reduce treatment-related toxicity. Curcumin serves as a representative example. Multiple studies have shown that curcumin induces apoptosis in human myeloid leukemia cells. Furthermore, metabolites derived from fungi and marine organisms, such as *Bifidobacterium longum*, have been shown to synergize with anti-PD-L1 immunotherapy to enhance antitumor efficacy (Sivan A et al., 2015). Recent research has further expanded the concept of natural products. This expanded definition now includes exosomes and Traditional Chinese Medicine-derived carbon dots (TCM-CDs), which exhibit high cellular uptake efficiency and excellent biocompatibility (Xin et al., 2024).

While extensive research has demonstrated the regulatory roles of natural products in the BME, their underlying chemical basis has not yet been systematically defined. Key mechanisms, including structure–function relationships, metal ion regulation, and redox modulation, remain fragmented and insufficiently integrated. Additionally, many natural products exhibit limited bioavailability in clinical applications, which substantially constrains their targeted therapeutic efficacy. Therefore, elucidating the chemical basis of natural products within the BME and developing more efficient extraction strategies, chemical modifications, and targeted delivery systems, is of significant importance.

**FIGURE 1:**
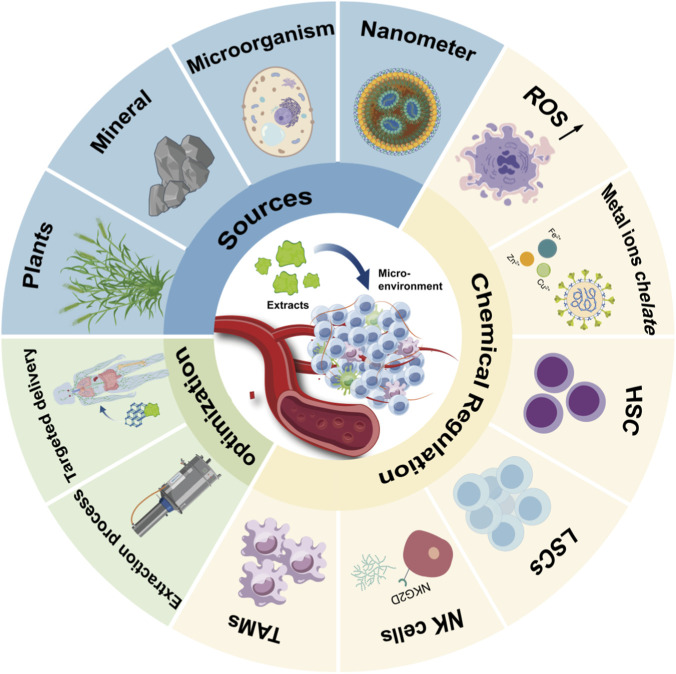
Full diagram illustrating the regulation of the blood microenvironment by natural products. TAMs, tumor-associated macrophages; NK cells, natural killer cell; LSCs, leukemic stem cells; HSC, hematopoietic stem; ROS, reactive oxygen species.

In this review, we focus on the chemical regulatory mechanisms of natural products in the BME. We systematically integrate their chemical characteristics with their biological effects, summarizing recent advances in extraction methods, chemical modification, and targeted delivery strategies. Finally, we discuss current challenges and future directions for the application of natural products in hematological therapy. Figure 1 illustrates the basic framework of this paper.

## 2 Core chemical mechanism

### 2.1 Redox and metal homeostasis regulation

The redox network comprises reactive oxygen species (ROS), reactive nitrogen species (RNS), and metal ion-medicated redox reactions, including those involving Fe^2+^/Fe^3+^ and Cu^+^/Cu^2+^. These species include singlet oxygen, superoxide anion (O_2_·^-^), hydrogen peroxide (H_2_O_2_), hydroxyl radicals (•OH), nitric oxide (NO•), and its highly reactive derivative peroxynitrite anion (ONOO^−^). Many of these potent oxidizing agents possess unpaired electrons, such as radicals like O_2_·^-^ and OH·^-^, which are characterized by atoms or molecules with one or more unpaired electrons in their valence shell. They readily participate in electron transfer reactions with radical and non-radical species (Giorgio M et al., 2007). Chemically, O_2_·^-^ is generated through one-electron reduction of molecular oxygen (O_2_); H_2_O_2_ is formed through superoxide dismutation catalyzed by superoxide dismutase (SOD); •OH are predominantly generated through Fenton or Fenton-like reactions mediated by transition metals, particularly iron or copper; NO• reacts with superoxide (O_2_·^-^) to form ONOO^−^, which exhibits substantially greater oxidative and nitrative capacity than hydrogen peroxide (H_2_O_2_) (Giorgio M et al., 2007; Radi, 2004; Sies, 2017). The redox state acts as a key regulator of hematopoietic stem and progenitor cells (HSC/HSPCs). It also regulates bone marrow mesenchymal cells, platelets, endothelial cells, and other components of the BME. Thus, the redox network exhibits two major cyclical patterns: the generation, interconversion, and elimination of ROS/RNS, and metal ion-facilitated electron transfer reactions. Pathological stress disrupts these redox processes, including inflammation, malignancy, and chemotherapy. Redox imbalance amplifies redox-sensitive signaling pathways. This amplification disrupts systemic homeostasis and complicates BME regulation.

Natural products often regulate biological systems through free radical scavenging. Phenolic hydroxyl groups present in polyphenols and flavonoids can neutralize H_2_O_2_ and •OH (Rice-Evans et al., 1996) through hydrogen atom transfer (HAT) or single-electron transfer (SET) mechanisms, inhibiting membrane lipid peroxidationand reducing intracellular ROS levels (Shahidi and Zhong, 2010). Additionally, accumulating evidence suggests that reduced intracellular ROS levels enhance the self-renewal capacity of HSCs (Ito et al., 2004), indicating that free radical scavenging may indirectly promote stromal support and remodeling of the BME. Furthermore, the presence of •OH has been demonstrated to facilitate the extraction of hydrogen atoms from glycosidic groups. This process instigates DNA chain reactions, which have the potential to induce genomic instability, cellular mutations, or carcinogenesis. (Lobo et al., 2010). Against this backdrop, glycosylation has emerged as a critical structural determinant influencing redox balance. Glycosylation increases the aqueous solubility and chemical stability of flavonoids. For example, O-glycosides and C-glycosides show higher metabolic stability than aglycones. These forms also display better water solubility *in vivo*. However, glycosylation reduces electron transfer efficiency and slows free radical scavenging rates (Xie et al., 2022). Moreover, Glycosylation can reduce plasma protein binding affinity and membrane permeability, thereby altering bioavailability and bone marrow distribution (Xiao et al., 2009). Moderate glycosylation enhances *in vivo* stability, whereas excessive glycosylation significantly reduces intestinal absorption efficiency. Accordingly, structural modification of natural products requires a careful balance between enhanced stability and preserving intrinsic antioxidant activity.

Metal ion chelation is considered another important mechanism by which natural products influence the BME. Fe^2+^ generates harmful ROS in the presence of hydrogen peroxide via the Fenton reaction. Fe^2+^ also reacts with molecular oxygen, producing ferric iron (Fe^3+^) and O_2_·- (Cherrak et al., 2016). Beyond the Fe^2+^/H_2_O_2_ system, Fenton-like reactions mediated by other metal ions such as Fe^3+^ and Cu^2+^, similarly elevate ROS levels and contribute to the damage of BME (Scarano A et al., 2023). As previously described, structural motifs such as diphenols and phenolic hydroxyl groups can chelate metal ions, thereby inhibiting Fenton and Fenton-like reactions. This reduces •OH production and consequently suppresses ROS generation. This mechanism becomes especially important under pathological conditions. These conditions include ineffective hematopoiesis, chronic transfusion therapy, and chemotherapy-induced oxidative stress. In iron-overloaded cellular and mouse models, excess iron directly increases ROS levels in bone marrow cells. This increase has been demonstrated to result in impaired HSC/HSPC function. Research findings have also indicated the upregulation of NOX4 and the activation of p38 signalling. However, antioxidants and the iron chelator deferoxamine can reverse these effects. This reversibility indicates a central role for iron-driven oxidative stress in hematopoietic suppression (Chai et al., 2015). Further mechanistic studies show that iron overload disrupts normal hematopoiesis through mitochondrial ROS accumulation, including the SIRT3-SOD2-mROS pathway. These findings highlight a close link between iron metabolism and hematopoietic imbalance (Zhou et al., 2021). Clinical evidence supports this mechanism. Patients with high-risk myelodysplastic syndrome (MDS) often develop iron deposition. This condition results from long-term transfusion, hematopoietic failure, or abnormal ferritin metabolism. Iron accumulation activates ROS-related apoptotic signaling pathways such as Wnt/β-catenin signaling (Huang et al., 2020). Similarly, iron chelators and antioxidants reduce the expression of related pathways, lowering intracellular ROS levels. In studies of the bone microenvironment, iron oxide nanoparticles enhanced the self-renewal and differentiation of hematopoietic stem cells by scavenging ROS and regulating metal ion homeostasis. This suggests that they may also possess similar regulatory potential in the blood microenvironment (Zheng et al., 2021). The high consistency between animal and clinical data supports the therapeutic value of metal-chelating and redox-modulating structures in natural products. These structures offer promising strategies for intervention in the BME.

**FIGURE 2:**
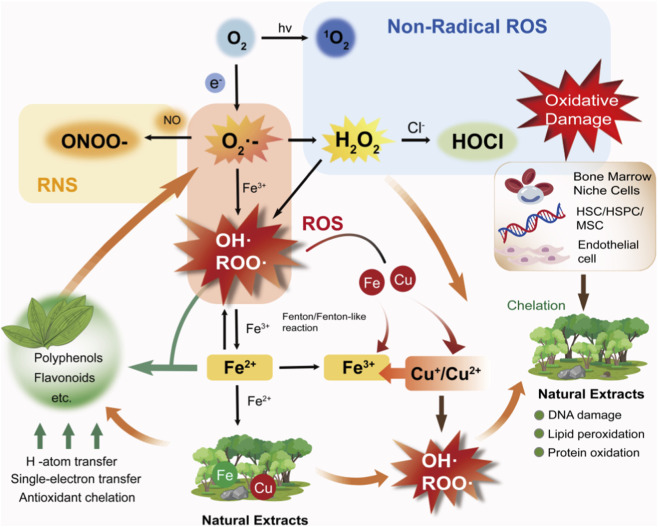
Schematic illustration of the dual regulatory roles of natural products and extracts in redox homeostasis and metal ion metabolism, and their impact on hematopoietic microenvironments. Natural products (e.g., polyphenols, flavonoids) scavenge ROS such as superoxide anion, ∙OH, and ROO∙ via hydrogen atom transfer and single-electron transfer, while also chelating labile iron (Fe^2+^/Fe^3+^) and copper (Cu^+^/Cu^2+^) ions to suppress Fenton/Fenton-like reactions and ROS generation. This dual action protects HSCs and BME from oxidative damage. Conversely, dysregulated metal ion pools and ROS accumulation can induce oxidative stress, DNA damage, lipid peroxidation, and protein oxidation, which contribute to leukemic stem cells (LSCs) survival and hematologic dysfunction.

Collectively, these findings highlight that redox modulation and metal ion homeostasis are fundamental chemical mechanisms of natural products. These mechanisms help preserve hematopoietic balance and maintain the integrity of the BME. Through these effects, natural products provide a chemical foundation for the regulation of intracellular signaling pathways. Figure 2 summarizes the basic chemical principles of this part and its effects on the hematopoietic system.

### 2.2 Chemical regulation based on covalent modification of signaling pathways

Natural products regulate redox balance and metal ion homeostasis through multiple chemical mechanisms. Many compounds act through nucleophilic, electron-donating groups, such as phenolic hydroxyls. Other compounds act through irreversible covalent interactions with target proteins. These interactions occur at specific active sites or amino acid residues. Reactive electrophilic moieties mediate this process. These electrophilic groups are commonly referred to as covalent warheads and can thus exert sustained inhibitor-like effects (Gersch et al., 2012). Typical covalent warheads can be classified as follows:

α,β-unsaturated carbonyl compounds possess a characteristic conjugated system formed by the carbonyl group and the adjacent carbon-carbon double bond. This structure imparts a pronounced electrophilicity to the β-carbon, which is highly susceptible to nucleophilic attack, particularly by the thiol groups of cysteine residues in proteins. The reaction facilitates Michael addition, resulting in the formation of stable C-S bonds (An et al., 2022; Sivan A. et al., 2015).

Epoxides: The oxygen-containing three-membered ring structure creates inherent strain, making it susceptible to nucleophilic attack and ring-opening reactions (Titov et al., 2011). Triptolide, which contains both an α,β-unsaturated carbonyl group and an epoxide moiety, has been shown to exert anti-inflammatory and antitumor effects. Several alkaloids have been reported to exert their biological effects through this mechanism (Herault et al., 2024).

Natural products can exploit covalent moieties to access and occupy the active pockets of target proteins, thereby modulating disease-related signaling pathways. The NF-κB signaling pathway is an example, impacting multiple hematological disorders. Particularly in leukemia, NF-κB signaling promotes the survival of LSCs and contributes to the establishment of an immunosuppressive microenvironment. Withaferin A (WA) is a naturally occurring electrophilic compound from Solanaceae plants. WA contains an α,β-unsaturated carbonyl group and an epoxide ring, enabling covalent binding to nucleophilic residues in proteins. Accumulating evidence indicates that WA inhibits NF-κB signaling by targeting the key kinase IKKβ, through covalent modification of the critical cysteine residue (Cys179) in its catalytic domain. Concurrently, its α,β-unsaturated carbonyl group may synergistically modify the NF-κB p65 subunit. In leukemia, such covalent chemical interventions have been shown to induce apoptosis in LSCs and attenuate the pro-tumorigenic effects of bone marrow stromal cells and macrophages (An et al., 2022).

### 2.3 Chemical regulation of key cells in the BME

#### 2.3.1 Selective regulation of HSCs and LSCs by natural products

The therapeutic potential of natural products arises from their complex, bidirectional effects on microenvironmental cells, particularly evident in HSCs and LSCs. HSC homeostasis depends on redox balance and feedback from signaling pathways such as hypoxia-inducible factor-1α (HIF-1α). On one hand, the scavenging of O_2_·-, •OH, and lipid peroxyl radicals suppresses lipid peroxidation, thereby mitigating damage to HSC membranes and nucleic acids. On the other hand, metal ion chelation markedly reduces labile metal pools, thereby limiting radical generation and decreasing intracellular ROS levels. Additionally, the stability of HIF-1α is a critical determinant of HSC function. Forristal et al. demonstrated that genetic deletion of HIF-1α in hematopoietic stem and progenitor cells impairs their response to granulocyte colony-stimulating factor. This defect leads to insufficient HSC mobilization (Forristal et al., 2015).

Under normoxic conditions, prolyl hydroxylase domain (PHD) enzymes hydroxylate specific proline residues on the HIF-α subunit, targeting it for degradation. Pharmacological inhibition of PHDs blocks this hydroxylation, stabilizing HIF-1α and promoting HSC proliferation, self-renewal, and bone marrow homing (Forristal et al., 2015). Multiple studies have demonstrated that metal chelators or natural products containing chelating moieties can further modulate HIF-PHD axis stability. For instance, the iron chelator deferoxamine (DFX) stabilizes HIF-1α by limiting Fe^2+^ availability required for PHD activity or by activating ROS-related signaling pathways. These effects highlight the therapeutic potential of metal chelation in hematopoietic recovery (Zhang X. et al., 2023). The plant-derived polyphenol quercetin, which contains hydroxyl functional groups, has been experimentally shown to chelate iron and inhibit PHD activity, thereby activating the HIF-1α-VEGF signaling pathway (Jeon et al., 2007).

Notably, HIF-1α homeostasis appears to exert bidirectional regulatory effects on the hematopoietic system. Wang et al. reported that, in AML mouse models harboring MLL-PTD and FLT3-ITD mutations, single-gene knockout of mutant HIF-1α impaired hematopoietic reconstitution during bone marrow transplantation. Combined genetic deletion produced a different outcome. Simultaneous knockout of HIF-1α with either Cited2 or von Hippel-Lindau (VHL) restored hematopoietic function. (Wang et al., 2014). These findings suggest that excessive HIF-1α accumulation may be detrimental to HSCs, while complete deficiency also compromises hematopoiesis. Together, these observations suggest that HIF-1α activity requires precise control. Optimal regulation, rather than simple upregulation, supports HSC quiescence and promotes hematopoietic recovery after BME injury.

In contrast to HSCs, LSCs depend on the sustained activation of signaling pathways such as HIF-1α, STAT3, NF-κB, and Wnt/β-catenin, and are highly sensitive to covalent protein modifications. Studies reveal that HIF-1α inhibitors enhance apoptosis in c-Kit^+^Sca-1^+^ cells, i.e., LSCs. Furthermore, HIF-1α inhibition also eliminates LSCs in human and mouse AML models, exhibiting minimal toxicity toward normal HSCs. These findings confirm the essential role of HIF-1α in LSCs survival (Wang et al., 2011).

Electrophilic or chelating natural products may primarily affect the HIF-1α pathway in LSCs through dual mechanisms: first, by chelating Fe^2+^, thereby impacting HIF-PHD stability; second, by influencing intracellular ROS levels, which in turn modulate HIF activity and metabolic reprogramming. In AML, such interventions reduce HIF-1α levels or disrupt its signaling, inducing apoptosis in leukemic cell populations. Electrophiles contain electron-deficient atoms and electron-withdrawing groups. Common examples include α,β-unsaturated carbonyls, isothiocyanates, and aldehydes. These molecules covalently modify cysteine or lysine residues in proteins, acting through Michael addition or thiol modification. (Hahn et al., 2018; Liang et al., 2022). Further research revealed that WA competitively inhibits the ATP-binding site of IKKβ. This dual action suppresses NF-κB activation, a mechanism relevant to leukemia treatment and the induction of LSC apoptosis. These findings suggest a chemical basis and therapeutic potential for electrophilic natural products to induce LSCs apoptosis and promote differentiation.

In chronic myeloid leukemia (CML), calmodulin-dependent protein kinase II (CaMKII γ) demonstrates heightened activation within LSCs. The natural extract berbamine (BBM), a bis-benzylisoquinoline alkaloid, competitively inhibits the ATP-binding site of kinases due to its bisbenzylisoquinoline skeleton and rigid aromatic side chain. BBM inhibits CaMKIIγ through this interaction and downstream signaling pathways, including STAT3, NF-κB, and Wnt/β-catenin. As a result, BBM blocks key survival signals in leukemic stem cells (Gu et al., 2012; Meng et al., 2013).

In summary, natural products have a dual effect on HSCs and LSCs by regulating redox balance, metal ion homeostasis, and intracellular signaling pathways. On one hand, natural products scavenge reactive oxygen species and chelate metal ions, protecting HSCs and supporting their self-renewal. These actions also help maintain HSC homeostasis, allowing for more effective recovery of hematopoietic function. On the other hand, natural products have unique electrophilic motifs, including α,β-unsaturated carbonyl groups and certain alkaloid scaffolds. These structures can covalently modify or competitively inhibit key survival proteins in LSCs, such as IKKβ and CaMKIIγ. This interference ultimately induces apoptosis in LSCs. The cell type-specific regulation demonstrates the chemical foundation of natural products, supporting their use in treating hematologic diseases such as multiple myeloma and acute myeloid leukemia. Additionally, it provides insights for developing therapies that selectively target and eliminate LSCs while preserving normal HSCs.

#### 2.3.2 Immune microenvironment remodeling: NK cells and TAMs

Within the immune microenvironment, multiple immune cells interact. This review focuses on natural killer (NK) cells and macrophages. Studies extensively demonstrated that redox homeostasis and metal ions strongly regulate their functions, closely aligning with the primary action of the natural products discussed in this review.

NK cell dysfunction represents a common pathological feature of many hematological disorders. The activating receptor NKG2D plays a central role in this process. Oxidative stress, iron overload, and cytokine signaling strongly influence NKG2D activity. NK cell cytotoxicity depends on the NKG2D-NKG2D ligand axis (Herault et al., 2024). Oxidative stress reduces NKG2D expression on the NK cell surface. However, this reduction can be reversed by hydrogen peroxide scavengers (Peraldi et al., 2009), thereby suggesting that intracellular ROS levels influence NK cell cytotoxicity (An et al., 2022). As discussed earlier, flavonoids and polyphenols have been shown to possess potent antioxidant propertie. Many studies report their regulatory effects on NK cell function and NKG2D expression. For instance, quercetin promotes NK cell proportion and maturation by binding to the MYH9 protein. MYH9 is known to play a critical role in hematopoietic stem cell differentiation, while also influencing T cell motility and promoting NK cell cytotoxicity (An et al., 2022; Su et al., 2024). In previous study, treatment with quercetin (25 μM) significantly increased the proportion of terminally differentiated NK cells (CD27^−^CD11b^+^). Co-treatment with the MYH9 inhibitor brebisistat weakens this effect. These findings suggest that quercetin regulates NK cell differentiation through MYH9, although further studies are required to elucidate the underlying molecular mechanisms (Su et al., 2024). Resveratrol provides another example. Resveratrol increases NKG2D mRNA expression in a dose-dependent manner. The compound also enhances NKG2D surface expression. This activation triggers ERK1/2 and JNK signaling pathways, inducing perforin production and perforin-dependent cytotoxicity (Chia-Chen et al., 2008). Most studies suggest that natural products boost NK cell activity. However, there is limited evidence about their capacity to prevent oxidative modification of NKG2D. Thus, the preservation of receptor–ligand binding capacity remains an unresolved question.

Furthermore, clinical and mechanistic studies consistently demonstrated that iron overload profoundly alters NK cell metabolism and oxidative status. These changes reduce NK cell cytotoxicity. In certain contexts, they trigger the demise of NK cells. This ultimately impairs immune surveillance against abnormal hematopoietic clones or tumors in conditions like MDS, leukemia, or the tumor microenvironment (Hua et al., 2017; Yao et al., 2023). Both *in vitro* and *in vivo* studies provide supportive evidence. Deferoxamine (DFX/DFO) or natural polyphenols significantly reduce dietary non-heme iron absorption, thereby restoring or enhancing NK cell-mediated cytotoxicity (Buerkli et al., 2022). Iron overload impairs NK cell recognition and cytotoxic function through multiple mechanisms. Excess iron increases intracellular ROS levels and upregulates ferritin expression. Iron overload disrupts the IFN-γ/STAT1 signaling pathway, further promoting ferritinophagy and ferroptosis. (Jiang and Eliott, 2017; Sottile et al., 2019).

Tumor-associated macrophages (TAMs) play a central role in immune regulation and are broadly classified into M1 and M2 phenotypes. M1 macrophages are generally considered to exhibit antitumor activity, whereas M2 macrophages are characterized by immunosuppressive functions (Petty and Yang, 2019). In the BME of hematologic malignancies, including AML, CLL, and MM, a substantial accumulation of M2 macrophages has been observed. Spatial omics analyses provide additional insight. These studies revealed enhanced inflammatory responses in patients with aplastic anemia (AA) and in bone marrow failure mouse models, accompanied by macrophage polarization toward the M1 phenotype (Glass et al., 2025). Additionally, quercetin has been reported to modulate M1/M2-associated markers through inhibition of NF-κB signaling or regulation of antioxidant genes (e.g., HO-1 and NQO1). Metal ions have likewise been demonstrated to play a regulatory role in macrophage polarization (Tsai et al., 2021). Research reveals that LPS/IFNγ-induced M1 macrophages exhibit downregulated ferroportin (FPN) expression, whereas IL-10-induced M2 macrophages display FPN upregulation. The iron-releasing phenotype of TAMs can be effectively suppressed by iron chelators, ultimately inhibiting breast cancer cell metastasis. This mechanism is likely to be relevant to hematological malignancies as well (Mertens et al., 2016). However, direct mechanistic evidence remains limited regarding how natural products influence macrophage polarization.

Nevertheless, numerous natural products have been reported to modulate macrophage-associated signaling pathways, often exhibiting antioxidant, anti-inflammatory, and cytoprotective effects. Astaxanthin is a representative example. Its long-conjugated double-bond system, together with the active electrons in its ketone and hydroxyl groups, enables efficient free radical scavenging. In human monocytic leukemia-derived macrophages (THP-1), astaxanthin was shown to suppress the release of macrophage-associated inflammatory cytokines (IL-1β, IL-6, IL-8, and TNF-α) via inhibition of the NF-κB pathway. Additionally, cellular protection against oxidative stress was mediated through activation of p53 and inhibition of STAT3 signaling (Wu Y. et al., 2024). Szlasa et al. reported similar effects for other natural compounds. They demonstrated that 0.5 μM of betulin (BE), betulinic acid (BA), and their derivatives can modulate the interactions between IL-6 and IFN-γ. These compounds also suppress the expression of COX-2 and effectively reduce inflammation in macrophages bearing lymphoma, specifically P388D1 cells. (Szlasa et al., 2023). Notably, the study identified stabilization of HIF-1α as a key mechanism by which TAMs regulate the HSC microenvironment. This regulation is achieved through increased VEGF-A secretion from bone marrow macrophages, thereby enhancing HSCs responsiveness to G-CSF-induced mobilization (Bisht et al., 2019). Together, these findings suggest that chemotactic regulation extends beyond just macrophages. This regulation can reshape the BME, likely through altered interactions between macrophages, HSCs, and LSCs.

In summary, TAMs play a pivotal role in shaping the immune and hematopoietic microenvironment through their polarization states. Natural products modulate macrophage function primarily through redox control and the modulation of signaling pathways. These actions affect macrophage polarization and inflammatory responses. Chemotactic and phenotypic changes extend beyond just macrophages. These modifications can reshape the hematopoietic niche by altering the interactions among TAMs, LSCs, and HSCs. Together, these effects highlight promising therapeutic opportunities for treating hematologic disorders.

#### 2.3.3 Structural and interface regulation: matrix cells and endothelial cells

The homeostasis of the BME similarly relies on an intact fundamental structure centered on bone marrow mesenchymal stem cells (BM-MSCs) and sinusoidal endothelial cells (BMECs). BM-MSCs secrete CXCL12 along with inflammatory mediators and other factors. These signals influence how abnormal cells and the surrounding microenvironment affect HSCs (Kojima et al., 2011). Natural products like resveratrol, with its polyphenolic structure, can influence deacetylases such as SIRT1 (Hu and Li, 2019). This reverses the pro-inflammatory secretion of MSCs under pathological conditions, restoring their capacity to support normal hematopoiesis. Quercetin, mitigates oxidative damage and senescence in MSCs, preserving their numbers and functionality.

BMECs act as a dynamic interface between the bone marrow and blood microenvironments, regulating both material exchange and the adhesion and proliferation of blood cells. Ginsenoside Rg1 promotes adhesion and proliferation of endothelial progenitor cells while enhancing VEGF expression to stimulate angiogenesis (Shi et al., 2009). The flavonoid baicalin has been found to interact with the tight junction protein ZO-1, modulating BMEC tight junctions and increasing interfacial permeability. This offers a novel approach for synergistic drug delivery and enhanced drug concentration (Hisada et al., 2020).

Collectively, chemical intervention targeting MSCs and BMECs fundamentally constitutes systematic regulation of the microenvironment’s structural foundation. By modulating proteins, cellular transport, and material exchange, it influences the BME.

## 3 Classification of natural products

Natural products exhibit significant structural diversity and extensive biological activity. Their active components differ in type and concentration, with these variations closely tied to their sources. Extracts from different sources possess unique chemical structures and confer distinct advantages in modulating the BME. Based on their biological and non-biological origins, they are primarily classified into four categories: plant extracts, microbial and marine metabolites, mineral extracts, and nano-natural products.

### 3.1 Plant extracts

Plant-derived natural products constitute one of the earliest and most extensively investigated classes of bioactive compounds. They have been demonstrated to exert significant effects on circulating cells, coagulation factors, and the immune system within the hematologic system. Active compounds such as flavonoids, polyphenols, alkaloids, and saponins can modulate the biological molecular environment through multiple mechanisms while demonstrating low toxicity.

**FIGURE 3:**
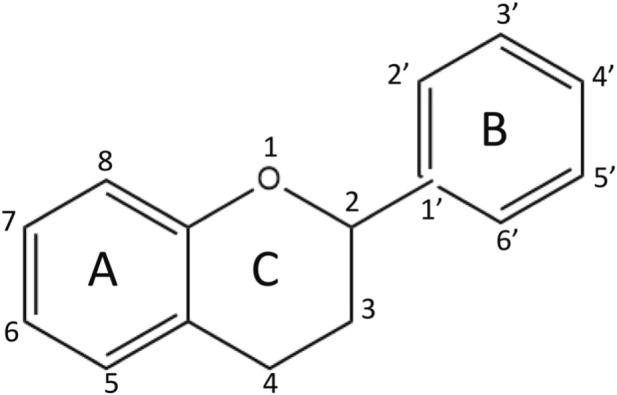
Chemical structure of flavonoids.

Flavonoids possess a characteristic C6-C3-C6 backbone (Figure 3 Chemical Structure). This core structure provides flavonoids with strong radical-scavenging and metal-chelating abilities, enabling them to regulate the BME (Farkas et al., 2004). This skeleton is also influenced by the number and position of hydroxyl groups (Nam et al., 2020). For example, fisetin is a natural tetrahydroxyflavone that significantly enhances hematopoietic function in mice exposed to cobalt-60 gamma radiation. This compound promotes the recovery of hematopoietic stem and progenitor cells. It also supports the repair of the BME. These findings highlight the therapeutic potential of flavonoid-based structures for targeted protection of the hematopoietic system (Liu et al., 2017). Additionally, the 3′,4′-dihydroxyl groups on the B ring are crucial for antioxidant activity. In contrast, glycosylation at the C3 hydroxyl significantly affects water solubility and bioavailability. Therefore, this position is an important target for chemical modifications during drug optimization. (Mishra et al., 2003; Xiao et al., 2009).

Alkaloids contain characteristic nitrogen atoms that mimic endogenous ligands, binding to protein receptors or ion channels to regulate vascular function and neurotransmitter release (Marileo et al., 2024). The representative compound vincristine is a classic chemotherapeutic agent for treating hematological malignancies such as acute lymphoblastic leukemia and lymphoma. The nitrogen atom of the indole ring and the rigid dimeric structure form the chemical basis for its binding to tubulin, inhibiting tubulin polymerization and ultimately halting cell mitosis (Paul et al., 2023). These findings indicate that compounds with complex alkaloid structures possess significant therapeutic value. These compounds can effectively target the proliferation of malignant cells and alter the cellular makeup of the BME.

**FIGURE 4:**
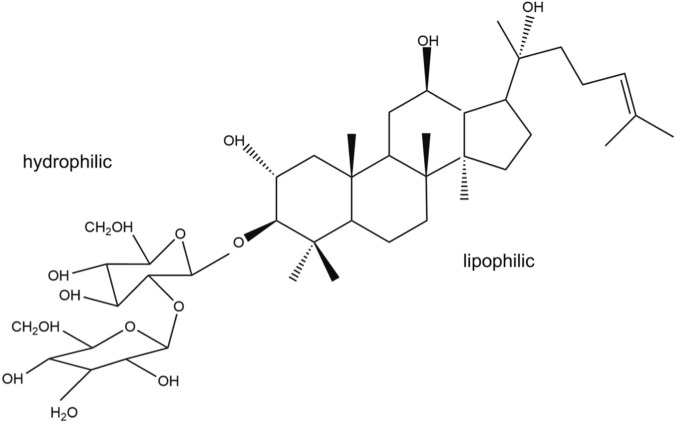
Amphiphilic structure of saponins.

Saponins exhibit a typical amphiphilic structure, composed of hydrophilic sugar chains conjugated to lipophilic steroidal or triterpenoid aglycones (Figure 4 Chemical Conformation). This amphiphilic design enables saponins to integrate into cell membranes, affecting membrane fluidity and stability. Consequently, saponins impact immune cell activation and cholesterol uptake. (Lorent et al., 2014).

Table 1 summarizes the key structure-activity relationships of common plant extracts. These relationships form a medicinal chemistry basis for multi-target and multi-level regulation of the BME.

**TABLE 1:** Characteristic structure and function of common plant extracts.

Name	Botanicalm source (representative)	Structure features	SAR	References
Flavonoids	Citrus, Ginkgo biloba, Tea	C6–C3–C6 skeleton with common hydroxyl, methoxy, glycosyl, or methyl substitutions	1. Polyphenolic hydroxyls and conjugated π system confer potent free radical scavenging and metal chelating ability 2. The position/number of hydroxyl groups is closely related to antioxidant capacity 3. Glycosylation affects solubility and bioavailability	Safe et al. (2021), Xie et al. (2022)
Polyphenols	Green tea, Grape seeds, Berries	Multiple phenolic hydroxyl groups	1. Redox activity depends on number and distribution of phenolic hydroxyls 2. Capable of non-covalent interactions with proteins/enzymes	El-Saadony et al. (2024)
Saponins	Ginseng, Quinoa, Licorice	Amphiphilic structure	1. Alters cell membrane permeability. 2. Sugar chain modulates affinity and solubility	Rojewska et al. (2023)
Alkaloids	Cephalotaxus harringtonia, Aconitine, Coffee	Alkaline nitrogen; Diverse skeletons	1. Basic nitrogen affects solubility 2. Receptor selectivity	Romero et al. (2019)
Terpenoids	Conifers, oaks, poplars	1. Isoprene (C5) unit carbon skeleton 2. Oxygen-containing functional groups	Determine covalent/non-covalent protein binding and activity	Masyita et al. (2022)

SAR, structure–activity relationship.

### 3.2 Mineral extracts

Active extracts derived from minerals primarily consist of inorganic compounds, trace elements, and their derivative complexes. These extracts are centered around metal ions and mineral matrices. They are essential for hematopoiesis and additionally contribute to hematologic homeostasis through ion exchange and chelation. Their chemical basis predominantly involves redox reactions, ion homeostasis, the modulation of enzyme activity and signal transduction, thereby playing a fundamental role in hematologic regulation.

Chemically, mineral extracts predominantly contain metal ions, including Fe, Zn, Cu, and Se, which serve as active centers. These metal ions are pivotal in mediating electron transfer within the hematopoietic microenvironment. For instance, Fe^2+^/Fe^3+^ and Cu^+^/Cu^2+^ participate in ROS generation through Fenton reactions (Chen, 2019). In contrast, Zn^2+^ and Se compounds act as cofactors for antioxidant enzymes such as SOD and GPx, maintaining redox homeostasis and protecting hematopoietic cells from oxidative damage (Huang et al., 2022). The mineral matrix has been shown to influence signaling pathways within the BME. Studies have demonstrated that iron deficiency impairs CD8^+^ T cell responses to infections or tumor antigens by suppressing the mTOR signaling pathway (Teh et al., 2025). Similarly, deficiencies in Zn and Mg can induce thymic alterations, thereby increasing the risk of lymphoproliferative disorders and leukemia (Lan et al., 2024). While the complete mechanisms are still not fully understood, these findings emphasize the significance of mineral metabolism. Maintaining mineral balance is essential for the metabolic stability and cytotoxic function of T cells. Furthermore, mineral extracts have been shown to exert significant effects on coagulation. Research has indicated that exogenous Zn^2+^ ([Zn^2+^]_o_) stimulates platelets, triggering Zn^2+^ influx and enhancing platelet activation (Kuravi et al., 2023). This process coincides with the phosphorylation of β3 integrin. These changes indicate that Zn^2+^ promotes platelet aggregation by regulating integrin conformation and activation on the platelet membrane (Feng et al., 2017).

Through characteristic ion regulation, redox, and signaling mechanisms, mineral extracts play a fundamental role in preserving the BME, modulating immune responses, and regulating coagulation. These insights provide both chemical and biological evidence supporting mineral-based hematopoietic and immunomodulatory research.

### 3.3 Microorganisms and marine metabolites

Natural products derived from microbial and marine sources primarily include polysaccharides and their related derivatives. These compounds often originate from fungi and bacteria, while marine sources also provide bioactive peptides, polysaccharides, and other metabolites (Pan et al., 2019). β-Glucan is a representative example of a glucose polymer derived from bacteria and fungi. It features a β-(1,3)-D-glucopyranose backbone, which allows for recognition by Dectin-1 and CR3 on macrophages and dendritic cells (Adams et al., 2008; Taylor et al., 2007). This interaction enhances antigen presentation and T cell activation, thereby contributing to immune regulation within the hematopoietic microenvironment. Furthermore, studies have indicated that β-glucan can modulate macrophage inflammatory responses via NF-κB or MAPK signaling pathways, reducing inflammation within the BME (Bergandi et al., 2021). However, Huang et al. reported that, in macrophages, β-glucan-mediated Dectin-1 activation is not associated with changes in the NF-κB pathway. Instead, it activates the downstream Syk-JNK-AP-1 signaling pathway within lipid rafts, promoting pro-inflammatory cytokine production and immune responses. These findings suggest that the role of β-(D)-glucan in the BME depends on the specific context. Receptor cooperation likely influences its effects, and the organization of lipid rafts also plays a role. Additionally, local inflammatory conditions can further affect outcomes. Together, these factors help maintain a balance between inflammation and hematopoietic homeostasis through either parallel or cooperative signaling pathways.

Molecular weight differences also affect bidirectional regulation of the coagulation system. Multiple studies have indicated that high-molecular-weight fucoidan exhibits potent anticoagulant activity, whereas low-molecular-weight fucoidan exerts anti-thrombotic effects (Manne et al., 2013). Medium-molecular-weight fucoidan has been suggested to possess superior anti-thrombotic activity compared with its low-molecular-weight counterpart (Zhao et al., 2012). This indicates that molecular weight not only determines a compound’s diffusion rate and half-life but may also modulate interactions with platelet, thrombin, and endothelial cell receptors.

Marine-derived peptides, polysaccharides, and their derivatives exhibit characteristic amino acid sequences that confer antioxidant, anticoagulant, and anti-inflammatory properties. For instance, studies have indicated that the Glu-Leu dipeptide is associated with antioxidant and anti-inflammatory effects (Hwang et al., 2012; Liu et al., 2020). Asp-Asn has been suggested to participate in metal ion chelation (Lu et al., 2024). Peptides that contain the Arg-Gly-Asp motif can inhibit cell adhesion and aggregation by recognizing integrin receptors (Lu et al., 2024). This suggests that specific amino acid sequences play a crucial role in reducing oxidative stress, maintaining ion balance in hematopoietic cells, and regulating coagulation.

In summary, natural metabolites demonstrate consistent structure-function relationships in the regulation of the BME. First, specific backbone structures play a crucial role in recognizing and binding to receptors or proteins, such as Dectin-1, selectins, and integrins, which subsequently activate downstream immune and hematopoietic signaling pathways. Second, molecular weight and sulfation levels facilitate bidirectional modulation of the coagulation system. Third, many compounds contribute to the protection or remodeling of the BME via antioxidant activity, metal ion chelation, or additional mechanisms.

### 3.4 Nano-sized natural products

In recent years, nano-derived natural products have emerged as innovative strategies for targeting the blood microenvironment. These materials can extend circulation time in the body, improve tissue penetration and retention, and be readily taken up by diseased cells (Xu et al., 2017). These agents primarily comprise exosomes, plant-derived nanovesicles (PDNVs) and their derived carbon dots (Table 2).

**TABLE 2:** Recent studies on the association of exosomes and carbon dots with hematologic diseases.

Source	Disease	Mechanism	Effect	References
AML-derived bone marrow mesenchymal stem cells	AML	Overproduction of ROS promotes exosome biogenesis and selective loading of miRNAs	Reduced lipid reactive oxygen species, disease progression, and drug resistance	Fan et al. (2025)
Exosomes loaded with gingerol and atractylodes lactone from human diffuse large B-cell lymphoma OCI-Ly3 cells	Diffuse large B-cell lymphoma	Inhibit NF-κB-dependent inflammation; Activate β-catenin-mediated crypt regeneration	Anti-tumor and alleviation of chemotherapy-induced diarrhea	Zhang et al. (2026)
MSC-derived exosomes	MDS	Exosomes rich in CPT-1A impair hematopoietic support function of MDS-MSCs	Reduces hematopoietic cell subsets and compromises MSC support for normal HSCs	Yin et al. (2025)
AML-derived exosome circ_006896	AML	circ_0006896 binds HDAC1, inhibiting lipid peroxidation-induced ferroptosis; also induces CD8^+^ T cell dysfunction via HDAC1/LEF1 axis	Promotes AML progression and impairs anti-tumor immunity; its inhibition may improve AML treatment	Can et al. (2025)
Donkey-hide gelatin Derived carbon dots (G-CDs)	AA	1. Promotes HSC proliferation/differentiation and erythrocyte maturation 2. Exhibits SOD-like and hydroxyl radical scavenging activity	Activates erythropoiesis, scavenges ROS, alleviates oxidative stress-induced apoptosis in blood cells	Wang C. et al. (2025)
Fenugreek seed PDNVs (FGDNVs)	Iron deficiency anemia (IDA)	Lysosomal degradation promotes iron release	Improve anemia symptoms	Bethi et al. (2025)
1. Exosomes derived from leukemic stem cells (LSCs) overexpressing RAB27B 2. Exosomes from LSC-derived exosome-educated senescent MSCs	AML	1. RAB27B enhances exosome secretion of senescence-associated proteins, preventing LSC senescence while inducing MSC senescence 2. Senescent MSCs feedback exosomes with stemness-maintaining proteins	Mediate the maintenance of LSCs through anti-aging effects and interactions with the microenvironment	Chen et al. (2024)

Exosomes are natural carriers in which internal proteins and nucleic acids are protected by a phospholipid bilayer (Del Pozo-Acebo et al., 2021). This structure supports blood stability and target specificity. Exosomal cargo is heterogeneous and comprises diverse proteins, lipids, and nucleic acids. These exosomes have been shown to modulate signaling pathways and immune homeostasis by influencing non-coding RNAs, metabolites, proteins, and other molecular components (Théry, 2011). For example, exosomes can decrease reactive oxygen species when stimulated by lipopolysaccharides (LPS). They accomplish this by activating the AhR-Nrf2 and Keap1-Nrf2 signaling pathways (Adamo et al., 2024; Urzì et al., 2023). Activation of the AhR pathway has been shown to reverse Th17/Treg imbalance in aplastic anemia (Guo et al., 2019). AhR is also involved in the regulation of immune and hematopoietic systems. Furthermore, exosomes may modulate leukemia progression via the AHR-ELMSAN1 axis (Zhou et al., 2025). Although systematic studies remain limited, these findings suggest that exosomes are capable of remodeling the BME through the regulation of key pathways.

PDNVs represent a unique class of extracellular vesicles that share essential structural and functional characteristics with exosomes. Each vesicle is composed of a phospholipid bilayer, which serves to protect the contents from degradation. This structure enables the vesicles to circulate stably in the bloodstream and facilitates their interaction with target cells in the BME. Their heterogeneous cargo, encompassing miRNAs, proteins, and bioactive metabolites, further underscores their potential to modulate hematopoietic and immune homeostasis (Del Pozo-Acebo et al., 2021). For instance, recent studies have demonstrated that the presence of phytoferritin nanocages in fenugreek seed PDNVs (FGDNVs) rescued iron deficiency anemia and restored hematological parameters. Thus, FGDNVs represent a natural iron nano-formulation for safe and efficient therapeutics for iron deficiency anemia (Bethi et al., 2025). Furthermore, the excellent ability of PDNVs in immune regulation makes them a promising tool for hematologic disease intervention. Collectively, both exosomes and PDNVs underscore the important role of extracellular vesicles in remodeling the BME. However, to fully achieve their clinical potential, challenges such as scalable isolation, standardization of cargo, and off-target effects need to be addressed.

Carbon dots are a novel class of photoluminescent nanomaterials exhibiting antioxidant, anti-inflammatory, hemostatic, real-time imaging, and tracking properties (Sharma et al., 2023). Their surfaces contain carboxyl groups, hydroxyl groups, and π-conjugated structures, which facilitate free radical scavenging and efficient electron transfer. These characteristics also contribute to their superoxide dismutase (SOD)-like catalytic activity. Furthermore, studies conducted in RAW264.7 cells have demonstrated that carbon dots possess antioxidant properties and the capacity to reduce ROS (Gao et al., 2023; Zeng et al., 2023). Xu et al. synthesized a novel carbon dot (J-CDs) based on jujube. Its structure contains sp^2^/sp^3^ carbon atoms and oxygen and nitrogen groups, exhibiting good biocompatibility and membrane permeability. J-CDs particularly promote the self-renewal of erythroid progenitor cells and further enhance erythropoiesis. This effect occurs through the regulation of hypoxia-response pathways and an increase in STAT5 phosphorylation. Safety studies demonstrate clear advantages. J-CDs do not promote tumor cell proliferation or metastasis, which contrasts with the effects of the control drug erythropoietin (EPO). These results suggest that J-CDs have strong potential for treating anemia related to cancer (Xu et al., 2022). Wang et al. further validated these principles. Their study revealed that Donkey-Hide Gelatin-Derived Carbon Dots (G-CDs), enriched with carboxyl, hydroxyl, and π-conjugated structures, can alleviate systemic oxidative stress in aplastic anemia (Wang et al., 2025). Additionally, G-CDs also promote the growth of hematopoietic stem and progenitor cells, facilitating their differentiation and maturation into the erythroid lineage. These findings indicate that carbon dots may regulate oxidative stress signaling and directly target the hypoxia-STAT5 signaling axis. This dual mechanism could enhance anemia treatment while minimizing tumor-promoting risks. Direct evidence for these mechanisms remains limited. Future studies should clarify how natural carbon dots interact with critical features of the pathological blood microenvironment.

The nanoscale structure of carbon dots enables efficient metal ion exchange and supports effective scavenging of free radicals. Consequently, nanoparticle-based natural products demonstrate significant regulatory potential in BME. These materials help maintain redox balance, suppress excessive immune activation, and promote tissue regeneration within the BME.

## 4 Research, application, and optimization of targeted effects of natural products

Natural products are widely utilized due to their chemical diversity, safety, and biological activity. However, their application is frequently limited by low targeting efficiency and poor bioavailability, thereby restricting therapeutic efficacy and clinical translation. Researchers consider enhancing the targeting ability of natural molecules essential. Structure-activity relationships (SAR) guide this improvement, while process optimization produces more stable active components. Carrier systems further enhance delivery efficiency, and surface modifications improve targeting performance.

Traditional extraction methods include maceration, Soxhlet extraction, and distillation. These techniques are easy to operate and require readily available equipment. Researchers continue to use them extensively in Chinese herbal medicine and the processing of natural extracts. However, their inherent limitations are increasingly evident. Maceration typically requires prolonged static incubation at room temperature following solvent addition, necessitating frequent agitation. As a result, this method is time-consuming and frequently leads to incomplete extraction (Shikov et al., 2022). The Soxhlet method is prone to degrading heat-sensitive bioactive compounds like polyphenols and flavonoids (Antony and Farid, 2022). Distillation is effective for the extraction of volatile compounds and essential oils. However, elevated temperatures may alter chemical composition and compromise bioactivity (Pheko-Ofitlhile and Makhzoum, 2024). Consequently, a series of advanced extraction techniques have emerged in recent years, including ultrasonic-assisted extraction (UAE), microwave-assisted extraction (MAE), enzyme-assisted extraction (EAE), supercritical CO_2_ extraction (SFE), and deep eutectic solvent (DES) extraction. Ultrasonic cavitation is used in the UAE to enhance solvent-solid contact, which increases extraction efficiency and helps preserve heat-sensitive compounds. Studies indicate that this method yields a higher phenolic content and stronger antioxidant activity from orange peel extracts (Chemat et al., 2017). MAE offers advantages comparable to those of UAE, primarily due to its short heating time and concentrated energy. EAE effectively releases polysaccharides and macromolecules under mild conditions, preserving their structural integrity. However, high enzyme costs and limited scalability still hinder its broader application (Nadar et al., 2018). SFE exhibits high selectivity toward nonpolar and volatile compounds, particularly terpenoids and steroids. Research indicates pressure plays a critical role in SFE: low pressure favors monoterpene extraction, while high pressure enhances extraction of sesquiterpene-rich compounds (Jokić et al., 2022). DESs represent a new class of green solvents. Research have indicated that DESs exhibit excellent extraction performance for flavonoids, polyphenols, alkaloids, anthraquinones, polysaccharides, and natural pigments. In many instances, DESs outperform traditional systems like ethanol or methanol (Li, 2022).

Based on the aforementioned extraction strategies, current optimization efforts are primarily focused on three key aspects: (i) optimization of extraction parameters, (ii) development of integrated extraction processes; for example, a study employing UAE-MAE synergistic extraction strategy. This approach leveraged the strengths of both methods, reducing the degradation of bioactive compounds. Additionally, it enhanced the phenolic extraction from various plants, including *Origanum vulgare*, *Rosmarinus officinalis*, *Hypericum perforatum*, and *Matricaria recutita*. (iii) extraction design tailored for targeted applications. Solvent choice plays a key role in this design. The solvent affects loading efficiency, particle size, and stability in delivery systems such as liposomes, nanoparticles, and exosomes (Laina et al., 2024). Polar solvents, such as water, are effective at extracting polar compounds, including polysaccharides and alkaloids. In contrast, nonpolar solvents like hexane and chloroform are used to extract lipophilic constituents (Beck-Broichsitter, 2021; Jovanović et al., 2025). Specialized delivery systems enhance this strategy by using therapeutic deep eutectic solvents (THEDES) formed from drug-DES combinations. These systems enhance stability and targeting by carefully selecting the appropriate solvents (Wu et al., 2021).

Effective delivery to the BME requires two complementary strategies: one involving the intrinsic modification of natural products and the other focusing on the surface engineering of exosomes and nanoparticles. Natural products contain reactive functional groups and characteristic scaffolds, including phenolic hydroxyl, carboxyl, and amino groups. Chemical reactions allow for site-specific modification of these groups, such as through esterification and etherification. It has been demonstrated that direct esterification of polyethylene glycol succinate with curcumin, mediated by N, N′-dicyclohexylcarbodiimide, enhances solubility and stability (Yakub et al., 2022). The previously described bis-BIA scaffold of BBM has also been systematically investigated. Electrophilic/hydrophobic substitution at the benzyl or aromatic acyl site (e.g., 2-methylbenzoyl modification forming BBD24) was found to confer enhanced cytotoxicity (Meng et al., 2013). Xie et al. further analyzed BBM derivatives with different substituents. Derivatives with aromatic groups at R1, R2, or R3 showed higher activity than those with fatty chains. Compound 2c carried an aromatic group at R1. This compound showed stronger activity (IC50 = 0.80 μM) than compound 2a with a fatty chain (IC50 = 3.14 μM). Among etherified BBM derivatives, electron-withdrawing groups enhanced activity. Compound 2e contained a nitro-substituted benzene ring at R1. This compound showed the highest cytotoxicity (IC50 = 0.36 μM). In contrast, esterified BBM derivatives responded differently. Electron-donating substituents on the benzene ring increased activity in this group. These findings highlight the value of structure-guided modification. Rational chemical design can significantly improve the biological activity of natural products (Xie et al., 2009).

Surface engineering enables precise delivery of nanocarriers. Zhang et al. developed a nanoprobe known as BP-PDA. This probe utilizes polydopamine (PDA) nanoparticles combined with graphene quantum dots (GQD). The system specifically targets immune checkpoint blockade in AML and facilitates real-time monitoring of immune responses. The nanoprobe achieves targeted specificity for AML cells. This targeting relies on surface conjugation with a LILRB4 monoclonal antibody. In the system, PDA serves as a fluorescence quencher, enabling a FRET pair with GQD. The FRET system enables real-time detection of granzyme B, a crucial marker of immune activation (Zhang D. et al., 2023). In addition, another study modified the surface of MgO nanoparticles with stearic acid (SA) and encapsulated them in PLGA microspheres. This enabled the carrier to stably release Mg^2+^ during degradation without causing drastic local pH fluctuations (Zheng et al., 2024). These findings demonstrate that surface engineering enhances the functions of nanocarriers. Additionally, surface engineering increases the precision of delivery within the BME. This approach represents a significant direction for advancing natural product delivery systems in the future.

The BME comprises multiple chemotactic and adhesion signaling pathways, among which the CXCR4-SDF-1 axis plays a pivotal role in HSC homing. Genetically engineered exosomes have been developed to enhance responsiveness to this signaling pathway. Hu et al. (2021) engineered donor cells to overexpress CXCR4 and collected their exosomes, delivering the miRNA antagonist (antagomir-188) to the bone marrow. This strategy significantly enhanced exosomal migration along the SDF-1 gradient toward the HSC niche (Hu et al., 2021), thereby improving homing efficiency. Other studies have confirmed the value of engineering CXCR4 on the surface. Luo et al. developed CXCR4-overexpressing vesicles that were loaded with miR-126. These vesicles targeted macrophages at inflammatory sites with high precision. The targeting resulted in the polarization of M1 macrophages to M2 macrophages (Luo et al., 2023). In addition to genetic engineering, chemical strategies have also been employed to achieve targeted delivery. The application of click chemistry reactions (CuAAC/SPAAC) enables site-specific modification of exosomal membrane proteins, facilitating the construction of chimeric antigen receptor-modified sEVs (CAR-sEVs) (Lu et al., 2025). Such engineered exosomes exhibit enhanced cell specificity and therapeutic efficacy, thereby increasing local retention of natural products and improving cellular uptake. Nanomaterials may utilize similar targeting principles. Nanoparticles can exploit chemotactic and adhesion pathways for active targeting. Carbon dots have abundant surface carboxyl and hydroxyl groups, which theoretically allow for ligand conjugation. However, experimental evidence remains limited. Overall, surface engineering improves targeting within the BME. Both genetic and chemical modifications play significant roles in this enhancement. These approaches provide promising pathways for the precise delivery of natural products.

## 5 Challenges and transformation path

Natural products have significant potential in BME. However, several challenges persist. Many underlying mechanisms remain poorly understood. Drug development and delivery face technical bottlenecks, and clinical translation remains difficult.

Key molecules like HIF-1α have bidirectional effects on HSCs and LSCs. Excess activation can be harmful, while complete inhibition also causes damage. Finding the right balance makes regulation challenging. Novel nano-natural products, such as carbon dots, promote erythroid differentiation without inducing tumor formation. Studies on J-CDs demonstrate significant effects on downstream genes of HIF-1α, including BNIP3, NDRG1, and PDK1. These effects likely indicate interactions between HSCs and LSCs (Wang et al., 2014; Xu et al., 2022). J-CDs may do more than activate hypoxia-responsive pathways and regulate the hypoxia-STAT5 signaling axis. However, the precise molecular targets of J-CDs within the BME remain unclear. Additionally, J-CDs seem to bypass HIF-1α-driven tumor-promoting pathways, suggesting a potential tumor-suppressive advantage. The role of natural products in immune regulation remains incompletely defined. Furthermore, several studies indicate that quercetin can enhance the function of NK cells. However, the specific molecular pathway that connects quercetin, MYH9, and NK cell differentiation remains unclear (An et al., 2022; Su et al., 2024). Additionally, there is a lack of direct evidence on how natural products prevent oxidative damage to NKG2D and maintain ligand binding. The regulation of macrophages shows several gaps. Iron overload affects macrophage polarization and function, while iron chelators can reverse pro-tumor macrophage characteristics. These discoveries emphasize the importance of metal ion homeostasis as a critical regulatory target in TAMs. However, direct evidence is limited on how natural products control macrophage iron metabolism, redox balance, and related signaling pathways. Additionally, the mechanisms that drive the polarization of macrophages toward the anti-tumor M1 phenotype remain unclear. A detailed elucidation of the molecular mechanisms is a prerequisite for designing enhanced anti-tumor immune surveillance therapies.

In extraction and targeted delivery, current optimization efforts primarily focus on improving yield. Techniques such as UAE, MAE, and DES rarely incorporate targeting strategies. Additionally, these methods often neglect chemical modification sites on active functional groups. Genetically engineered exosomes offer a novel approach. Click chemistry facilitates precise modifications to functional groups. This technique enables specific conjugation while preserving the integrity of the natural product backbone, ensuring that exosome activity is maintained (Lu et al., 2025). However, to ensure the precision of targeted delivery systems, the development of multi-response intelligent delivery systems similar to the BP-PDA system is still needed. By actively addressing these challenges and technological bottlenecks, natural products hold promise as a potentially widespread therapeutic approach.

## 6 Conclusion

Natural products provide distinct advantages in transforming the BME. These benefits stem from their excellent safety profile, multi-target effectiveness, and diverse chemical structures. Their characteristic chemical structures, including functional groups such as phenolic hydroxyl, carboxyl, and amino moieties, are responsible for antioxidant activity, metal ion chelation, and redox regulation. These physicochemical properties have been shown to influence the function of key cellular components, including HSCs/LSCs, NK cells, TAMs, and the bone marrow stroma. This review provides a systematic analysis of SAR in various natural products, explaining the chemical basis for BME modulation and identifying current gaps and ongoing controversies in the field.

Natural products have significant potential to reshape the BME field and treat hematological disorders. However, several limitations still hinder their application, including low targeting efficiency, poor bioavailability, and limited stability. In recent years, researchers have proposed many optimization strategies. Advanced extraction methods have improved both the yield and purity of substances. DES-assisted extraction has enhanced solubility and efficiency. Additionally, chemical modifications, such as esterification and click chemistry, have further refined molecular properties. Moreover, new delivery systems based on nanotechnology and exosomes have been developed to enhance targeting ability and tissue retention. However, challenges related to pharmacokinetics, safety, and interindividual variability remain to be fully addressed.
